# Yield improvement of *Gracilaria tenuistipitata* by optimizing different aspects in coast of cox’s bazar, Bangladesh

**DOI:** 10.1038/s41598-022-08040-3

**Published:** 2022-03-09

**Authors:** S. M. Bokhtiar, M. A. Ali, M. A. Z. Chowdhury, K. U. Ahmed, M. K. Hassan, M. Ahmed, M. S. Bhuiyan, O. F. Mashuk, M. M. Rahman, M. A. Salam, S. M. Rafiquzzaman, Md Faruque Hossain

**Affiliations:** 1 Bangladesh Agricultural Research Council, Dhaka, Bangladesh; 2 Bangladesh Agricultural Research Institute, Gazipur, Bangladesh; 3 Bangabandhu Sheikh Mujibur Rahman Agricultural University, Gazipur, Bangladesh

**Keywords:** Plant sciences, Ocean sciences

## Abstract

This research was designed to find out the effect of different factors such as influence of lunar cycle, harvesting interval, rope type and seeding gap on the production of *G. tenuistipitata* in coast of Cox’s Bazar. Duration of these experiments were sixty days and all the parameters were recorded fortnightly. Monitoring of water quality parameters indicated that salinity, temperature, transparency, pH and DO were suitable for seaweed cultivation. In determining lunar cycle effect, results envisaged that fresh yield was 14.43% increased when seeding and harvesting time was selected considering the moon cycle. Regarding the selection of harvesting interval, it was found that T_3_ (30 days interval) was the best to harvest the seaweed whereas T_4_ (40 days interval) showed decreasing trend in production. Our study also found that semi floating single line showed better yield performance compared to semi floating double line system. In case of influence on seeding gap, it has been found that 20 cm gap between two seed showed the highest yields followed by 10 cm, 30 cm and 40 cm, respectively. Overall, it can be concluded that yield of *G. tenuistipitata* in coast of Cox’s Bazar could be improved considering those factors.

## Introduction

Seaweed is a colloquial term for the common name of mostly macroscopic and multicellular marine algae, which do not have root systems or flowers, leaves, stems, fruits and seeds and generally grow and live attached to rock or other hard substrata below the high-water mark or remain drifted in the oceans^1–4^. Seaweeds regarded as a high profile commercial marine biota for its variety of uses, like raw materials of bio-chemicals (agar, agarose, algin, and carrageenan), dyes, food, feed, enzymes and drugs^5^. Bangladesh is in the transitional zone for the flora and fauna of the Indian subcontinent and Southeast Asia, and is part of the Indo-Burma biodiversity hotspot^6^. It has 710-km-long coastline and a 25,000-sq-km coastal area which support a variety of land use practices. The seacoast of Bangladesh is considered as one of the unreached areas of the world in the field of phycology. This coastal area, with both sandy and muddy beaches, estuaries and mangrove swamps, provides substrates and habitats for the cultivation of various kinds of seaweeds, according to experts. *Gracilaria* species are notable for their economic importance in the production of agar, human food, animal feed, fertilizer, drugs, and biofuel^7^. With a cosmopolitan distribution and high growth rates, this species is considered as good candidates for cultivation in various parts of the world^8^.

Seaweed culture in Bangladesh is still in an initial stage. However, a few researchers are involved in seaweed cultivation in the south-eastern and south-western coasts of Bangladesh and the methods of cultivation of seaweeds using indigenous materials like bamboo and rope^6^. There are a limited number of experiment conducted on culture of seaweed in Bangladesh where all of them either focused on the adaptation of culture technique based on environmental parameters or identifying potential sites for seaweed culture^9–11^. No experiment conducted regarding other possible factors which has influence on the yield performance of the seaweed culture in Bangladesh. Harrison and Hurd^12^ reported that success in cultivating seaweed depends on both of the knowledge of ecophysiological characteristics of the species and on the important factors for seaweed growth that are responsible for better yields performance.

Hence, the aim of this study was to observe the effect of some crucial factors such as lunar cycle, harvesting interval, planting system and seeding distance on the production of seaweed *Gracilaria tenuistipitata* in Nuniarchara coast, Cox’s Bazar.

## Results

### Water quality parameters

Seaweed culture needs suitable physico-chemical parameters. The mean (± SD) values of different water quality parameters during the experimental period are presented in Table 1.

**Table 1:** Mean values of water quality parameters during experimental period of 60 days.

Average water quality parameters					
Parameters	Salinity (ppt)	Temp. (°C)	Transparency (cm)	pH	DO (mg/L)
	32 ± 0.3	22 ± 0.1	74.5 ± 1.4	8.0 ± 0.1	7.2 ± 0.1

### Nutrient parameters

Nutrient parameters of water and soil in the seaweed beds of Nuniachara coast of Cox’s Bazar are presented in the Table 2; n = 3.

**Table 2:** Water and soil nutrients (mean ± SD) of seaweed bed in Nuniachara coast of Cox’s Bazar.

Water parameters	Unit
Nitrate (NO_3_) (mg/L)	0.632 ± 0.2
Nitrite (NO_2_) (mg/L)	0.443 ± 0.11
Sulphate (S) (mg/L)	10,234.25 ± 125.3
Calcium (Ca) (ppm)	437.30 ± 7.1
Soil parameters	
Soil calcium (Ca) (ppm)	16,387.32 ± 103.5

### Effect of lunar cycle on the yields of *Gracilaria tenuistipitata*

In this experiment in T_0,_ seeds of seaweeds were transplanted three days after full moon and harvested three days before next full moon. On the other hand in T_1,_ seeds were transplanted before full moon and harvested after that full moon. It is found that, full moon has impact on the yield performance of *Gracilaria tenuistipitata* (Table 3)***.***

**Table 3:** Effect of lunar cycle on the yield performance of *Gracilaria tenuistipitata.*

Treatments	Full moon during experiment	Fresh yield (t ha^-1^)	Dry yield (t ha^-1^)
T_0_	12 December and 11 January	9.12 ± 0.66	1.55 ± 0.19
T_1_		7.97 ± 0.80	1.35 ± 0.14
∆T = T_0-_T_1_		1.15	0.20
% increase		14.43	14.81

The yields found higher in T_0_ (where seeds were transplanted after full moon and harvested before the next full moon) than T_1_ (where seeds were transplanted before full moon and harvested after full moon).

### Effect of harvesting interval on the yields of *Gracilaria tenuistipitata*

**Figure 1:**
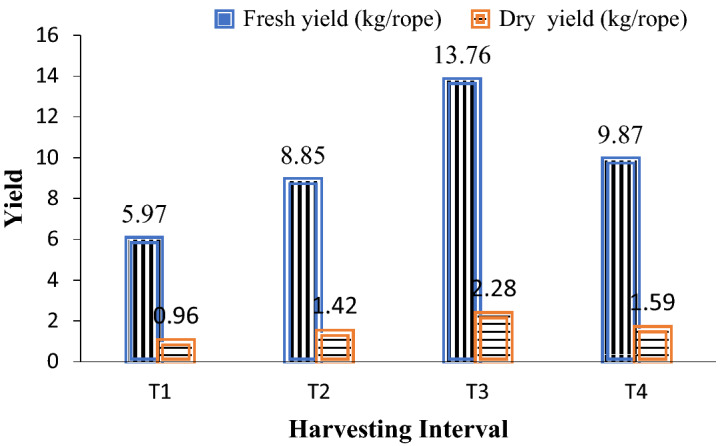
Effect of harvesting interval on the yields of *Gracilaria tenuistipitata.*

In this case, the impact of harvesting interval (10, 20, 30, 40 days) on the yield performance of *Gracilaria tenuistipitata* presented in Fig. 1.

It is found that the production increased in T_1_, T_2_ and in T_3_ where the yields were highest in T_3_ and the production decreased in T_4._ These results indicate that 30 days of interval are the peak period to harvest the seaweed.

### Effect of planting system on the yield

Two methods of planting system were followed to see the yield performance of *Gracilaria* species. It is found that the yield performance is better in semi floating single line (T_1_) than semi floating double line (T_2_) (Table 4 and 5).

**Table 4:** Yield performance of *Gracilaria tenuistipitata* in semi floating single line (T_1_).

No. of row	Fresh weight (ton/ha)	Dry weight (ton/ha)
1	12.78	2.03
2	13.63	2.18
3	11.99	1.92
4	13.52	2.16
5	11.79	1.87
Mean ± SD	12.74 ± 0.85^A^	2.03 ± 0.14^A^

**Table 5:** Yield Performance of *Gracilaria tenuistipitata* species in semi floating double line (T_2_).

No. of row	Fresh weight (ton/ha)	Dry weight (ton/ha)
1	7.49	1.29
2	6.92	1.15
3	7.91	1.19
4	6.75	1.09
5	7.31	1.24
Mean ± SD	7.28 ± 0.46^B^	1.19 ± 0.08^B^

The fresh yield in semi floating single line was 1.274 ± 0.08 kgm^-2^ that was significantly higher than the yield in semi floating double line system (0.728 ± 0.046 kgm^-2^).

### Effect of seeding distance on the yields of *Gracilaria tenuistipitata*

**Figure 2:**
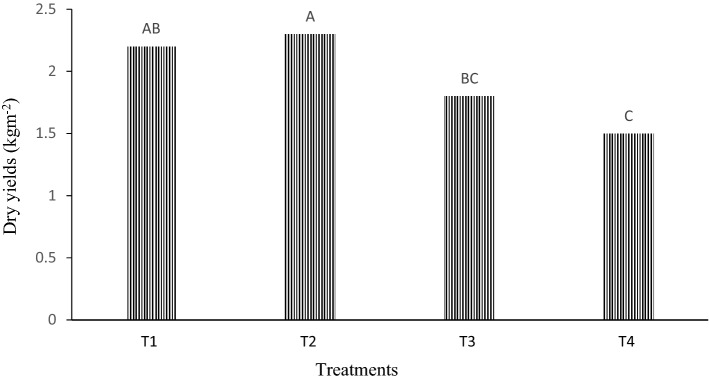
Dry yields in different seeding distance. Bars with different letters were significantly (p < 0.05) different from each other.

The yield of *Gracilaria tenuistipitata* seedlings in each treatments during the cultivation periods (30 days) is graphed in Fig. 2. It shows that the planting distances influence the yields of *Gracilaria tenuistipitata* (p < 0.05).

The 20 cm length of planting distance showed significantly higher amount of yields (0.23 kgm^-2^) than the other three treatments. T_4_, which denotes the planting distance 40 cm showed the lowest yield performance (0.15 kgm^-2^).

## Discussion

Seaweeds are not only source of food, feed and medicine but also a source of bioactive compounds which has nutritional and biomedical applications^13^. Considering the importance, seaweed cultivation has been an issue of much importance all over the world in recent years. *Gracilaria tenuistipitata* is an important seaweed species which has tolerant to a wide range of environments and an economically important raw material for agar production^14^. Most of the researches on seaweed farming is limited to searching the suitable location, selection of farming method and suitability of seaweed seed^10,11,15^. The success of seaweed farming does not only depend on ecological suitability of the site, but also to a large extent on some factors that also have impact on the production performance of seaweed. In this study, we evaluated influences of some other factors like effect of lunar cycle, rope type, harvesting interval and seeding gap on the improvement of production performances.

In these experiments, the water quality parameters and nutrient parameters of soil and water can be said suitable for seaweed culture according to other research findings^16–18^. *Gracilaria* has a high tolerance of environmental parameters^19–21^. The optimum environment could improve nutrient absorption process so that it could improve the growth rate of seaweeds^10,21,22^.

Regarding the effect of lunar cycle or full moon, seeding and harvesting was carried considering lunar cycle as tidal effect showed strong influence on the productivity of seaweed farming. It is worthy to mention here that during the full moon when both moon and sun are in a straight line on opposite sides of the earth which creates gravitational forces combine to create larger tidal bulges. So considering the tidal effect experimental design was made to get the optimum yield *Gracilaria tenuistipitata*. From the experiment, results showed that full moon has clear influential effect on the yield of *Gracilaria tenuistipitata* since during the day of full moon and three days before and after the full moon, the wave pressure remains higher than usual. Mulford^23^ reported that when the moon is full, this causes a stronger pull-on ocean as the Earth and Moon are aligned, which causes a more pronounced tide. In present study, reduced yield performance recorded when seaweeds were harvested and transplanted in between two consecutive full moons. On the other hand, better yield performance recorded when the seeds were transplanted three days after full moon and harvested three days before next full moon. This was happened because during full moon in the middle or late of cultivation cycle, highest high tide and waves occur. Stronger high tide reduced the yield by storming off the long crop rope of *Gracilaria tenuistipitata.* Yield may be increased by 10 to 15 percent if seeding is done three days before the full moon and harvest it three days after the next full moon (24 days field duration), instead of seeding at first day of the month and harvesting at last day (30 days field duration). Thus, we can save six days which can be used to dry and processing the harvest and also to take preparations for the next seeding. It has been found that harvesting duration also another factor which may influence on the productivity of seaweed farming. Timely harvesting of any crops ensures good crop quality and high market value. Another experiment also carried out to search out the suitable harvesting interval for obtaining better yield. In our present study, it is found that harvesting seaweeds after 30 days of seeding showed maximum yield performance. Reduced yield performance found when seaweeds were harvested after 40 days of seeding. The reason behind this could be rotting of the bunch of ropes of seaweed after 30 days of period and loss of the yield of seaweed has been occurred. Although limited research findings is available in the literature regarding this factor, our study revealed that 30 days of harvesting interval is the peak period to harvest. Padhi et al.^24^ in a study revealed that *Gracilaria verrucosa* reached harvestable size after 30 days and a maximum 15- and 13.8-fold increase in biomass in raft culture and rope culture respectively. It was also observed that production was increased 11.6- and 11.0-fold in biomass at Samal of Chilika Lake which is in agreement with our findings. Again, four folds increase in biomass was obtained after 25 days of seeding of a red seaweed species *Hypnea musciformis* in the lagoon of Krusadai Island, India on long line ropes^25^. Yang et al.^26^ reported an increase in biomass of *Gracilaria lemaneiformis* from 50 to 775 g FW m^−1^ at 28 day harvest intervals with, a specific growth rate of 13% day^−1^ using horizontal cultivation in Jiaozhou Bay, China which is also in line with our present study findings. Again, the initial stocking density of 25–50 g/m^2^ with a culture period of one month was found to be optimum by Sarkar et al.^27^ of extensive culture of *Gracilaria tenuistipitata* in pond system filled with natural estuarine brackish water throughout the year. Harvesting needs to be carried out in such a way as to maintain *peak* productivity. However, the duration of the period between planting and harvesting depends first of all on the growth rate and on the time when the seaweeds gain maximum commercial value such as high content of polysaccharides or certain taste properties^28^.

Rope type in cultivation method has significant impact on the yield performance of the *Gracilaria tenuistipitata.* Methods of seaweed cultivation are greatly varied according to various factors such as cultivation facilities (in the open sea or on the land), productivity and availability of species, dimensional characteristics of an aquatic ecosystem (size and depth) irradiance, temperature conditions, nutrient enrichment, water movement and degree of wave action^28^. Considering these factors we single line semi-floating method and double-line semi-floating method to produce *Gracilaria tenuistipitata* in sand-flat of Nuniarchara near shoreline in this study and also investigated in comparative suitability between two methods in response to biomass production, management suitability and cost effectiveness. From the literature it has been evident that *Gracilaria *sp. can be cultured in open sea using different cultivation techniques- coral stone method, concrete block method, single rope floating technique (SRFT), bamboo raft method and tube net method. Krishnamurthy et al.^29^ recorded 20 ton dry wt ha^-1^ year^-1^ of *Gracilaria edulis* following long-line rope method where vegetative apical fragments of 3–4 cm long were inserted between the braids of coir rope. Subbaramaiah and Thomas^30^ obtained more yield of *G. edulis* (30 t dry wt ha^-1^ year^-1^) following single rope floating raft technique where seaweed fragments were seede on several vertical lines of 1 m long ropes tied to 20 m long horizontal ropes. In this study we used single line and double line semi-floating method where seeds were seeded into 25 m long horizontal ropes by making hole using untwining the strands and then cultured in open sea. We opted this technique considering cultivation facilities, geographic and dimensional characteristics of our cultivation site. For double line semi floating method two adjacent single ropes were attached with bamboo sticks at gaps of 15 cm for better management practice comparing with single line method, since during cultivation period due to high wave pressure it was found that single ropes sometimes haphazardly lied on sand-flat. We used the term ‘semi-floating’ because some parts of a day the cultured ropes lied on exposed sand flat during low tide and most of the time remained hanging during high time. In our study, it has been seen that semi floating single line rope method was found better over semi-floating double line rope method and the yield was significantly higher in semi floating single line rope method. The higher yield was found due to fact that seeds of double line floating system were more submerged than the single line floating system and it caused to decompose the seeds of seaweeds and reduced the yield. Additionally, penetration of sunlight was less due to double line floating system which ultimately hampers the photosynthesis. Most of the researchers mainly focused on methodological approaches and main cultivation methods and discusses different problems arising during the application of these methods^28,31^ but no experiment found to be conducted regarding the planting method on the yield performance of *Gracilaria tenuistipitata.*

Seeding gap is a crucial factor for seaweed production. Neish^32^ reported that the suitable planting distance will provide wider water circulation which take nutrients leading to enhance diffusion process in upgrading the metabolic and growth rate. In our present study, 20 cm length of seeding gap resulted significantly higher amount of yields. In this study, the 20 cm-seeding gap is assumed to be suitable for receiving the intensity of sunlight in all thalli parts of *Gracilaria*, while in the other planting distance (10, 30 and 40 cm) might be too narrow or too wide that indicates thalli’s surface cover each other and also covered by filamentous epiphyte algae that impede sunlight absorption. These findings are in line with a report of Reddy et al*.*^33^ and Aslin et al*.*^34^ who found similar result in their experiment.

Taking all those factors into account, these studies revealed that *Gracilaria tenuistipitata* species could be cultured in our coast particularly in Nuniachara coast of Cox’s Bazar. However, for getting maximum production, some effects such as lunar cycle, rope type, harvesting intervals of the seaweed and seeding gap need to be considered as our studies finds that there are influential impact of this factors on the yield performance of *Gracilaria* species. So, further research is needed to find out the effect of various factors on the yield performance of seaweed.

## Methods

### Study area

**Figure 3:**
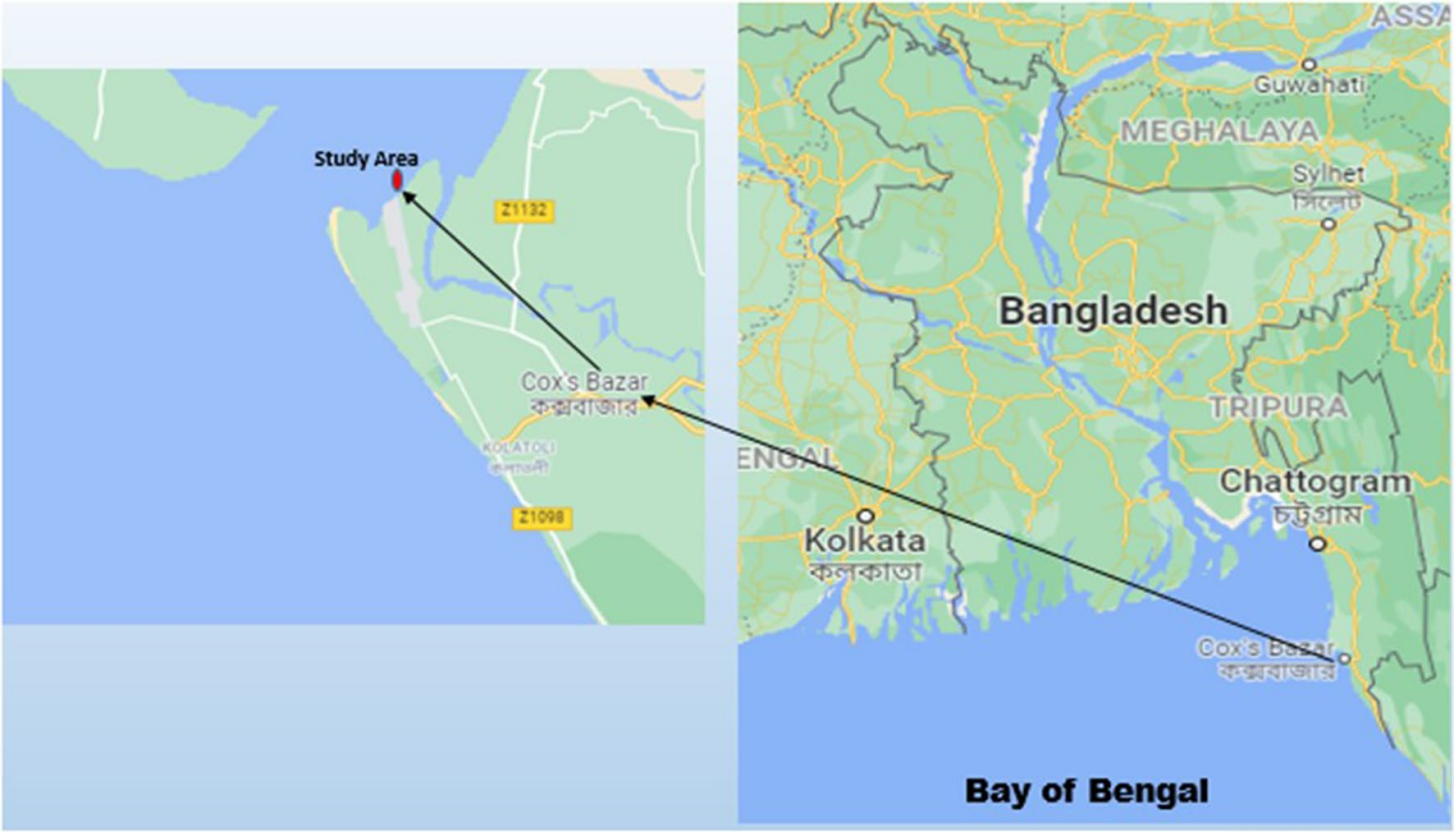
Google. (n.d.). [Location of the study area at Nuniarchara, Cox’s Bazar]. Retrieved November 15, 2021, from https://goo.gl/maps/pfx8rtfQR2ZxZ8GY9.

Study was conducted from December 2019 to January 2020 in Nuniachara of Cox’s Bazar sadar. The cultivation of seaweeds in open sea is being carried out using one-step seed production method which requires healthy seaweeds. Taking the above considerations into account, experiment site has been primarily selected at Nuniachara coast of Cox’s Bazar sadar. Nuniachara coast has 300 m East–west × 2000 m North–south sand-flat intertidal zones which is very suitable for off-bottom and Semi-floating methods having relatively low turbidity but regular about 2 m high tides. Natural seaweed beds are found at Nuniarchara to Nazirartek areas of Bakkhali River and Moheshkhali Channel estuary and in Moheshkhali Island. *Hypnea musciformis* and *Enteromorpha intestinalis* are the main seaweed species of seaweed beds^6^. Geographic position of the study area is 21°28′26.1''N and 91°57′51.5''E which lies on near shoreline and there is no coral reef and seagrass vegetation in and around the area (Fig. 3). Some marine aquatic plants are found growing on silted sand-flat during rainy season when salinity becomes lower.

### Collection of seaweed seed

Wild *G. tenuistipitata* as seed was collected from Saint Martin’s Island (92º28ʹ40.12ʺE and 20º65ʹ51.43ʺN) of Bay of Bengal of Bangladesh. The total area of the island is about 12 km^2^ with the island itself being approximately 5.9 km^2^ and the rest of the area being rocky platforms entering the sea (DOE, 2012)^35^. The key habitats of the islands are rocky and sandy inter-tidal habitats, rocky sub-tidal habitats, offshore lagoons, sea grass beds, coral aggregations, soft coral habitats and offshore soft-bottom habitats^36,37^. The dominant marine species are corals, reptiles, turtles, seagrass meadows, algal flora, gastropod-algae, molluscs, echinoderms, reef fishes and shorebirds^38^. Rocky substrates, which are crucially required for seaweed habitat are available around the St. Martin Island except the north coast (FAO/NACA, 1996). Water quality parameters of this island are very amiable for growth and seasonal abundance of different seaweed spices. Therefore, St. Martin Island is an extraordinary place for natural availability of seaweeds. Seed collection from seabed was permitted from the local government and authenticated the botanical identification of seaweed species as the voucher specimen has been previously deposited at BFRI herbarium.

### Production of seeds

The farming/cultivation of seaweeds in open sea was carried out using one-step seed production method. Seeds for seaweed farming was produced by one-step (directly using cut pieces and tied with the ropes) and multi-step (inducing spore release by a particular seaweed in the green house and allowing spores to grow on ropes and nets) methods.

### Seaweed culture method

The seeded ropes were arranged in single or double lines in the open sea. Each line was kept attached with two bamboo poles so that it could not be washed away. Plastic floats @ 5 pieces per 25 m long rope were used to keep the ropes moving up and downwards during tides. No fertilizer, insecticides and pesticides were used during seaweed cultivation.

### Water parameters

Water temperature, salinity, transparency, pH and dissolved oxygen (DO) were checked every 15 days at the cultivation sites. Transparency was measured using Secci disc. A digital DO meter (HACH, USA) was used to determine the dissolved oxygen content of water. pH was measured using a digital pH meter (HACH, USA). Salinity was determined using refractometer.

### Collection and analysis of soil

Soil samples from the sites were collected and after collection, samples were brought back to the laboratory within 6 h for analysis. In the laboratory, the samples were dried and powdered, sieved and kept in a desiccator until further analysis.

### Optimization of different factors

#### Experiment 1: effect of lunar cycle

This study was conducted to find out the effect of lunar cycle on the yield performance of *Gracilaria tenuistipitata.* In this experiment, T_0_ indicates control, where the seeds were transplanted three days after full moon and harvested three days before next full moon. Therefore, there was effect of full moon during cultivation period. On the other hand, T_1_ indicates that seaweeds were transplanted before full moon and harvested after full moon where one highest high tide occurred during cultivation. 25 m long ropes were seeded maintaining 20 cm seeding gap and placed on sand flat at a distance of 40 cm with the help of bamboo poles on first day of December 2019 and harvested on 31st December. Full moon occurred on 12th December. Average weight of each seed was 2 gm. Each rope was harvested separately and weighed with the help of digital balance. Fresh product was dried under sun for one whole day to get dry yield. On the other hand, another set of ropes were seeded and transplanted in open sea three days after the full moon which was on 15th December. Next full moon occurred on 11th of January 2020. To avoid effect of full moon ropes were harvested on 8th of January 2020. Fresh and dry yield was obtained in same way as stated for previous set of ropes. Plastic floats @ 5 floats/ rope were used to help the ropes floating during high tide.

#### Experiment 2: effect of harvesting interval

In this experiment, different harvesting intervals (T_1_ = 10 days, T_2_ = 20 days, T_3_ = 30 days, T_4_ = 40 days) were taken to determine the best harvesting time with *Gracilaria tenuistipitata* by harvesting after 10 days, 20 days, 30 days and 40 days of seeding, respectively for getting maximum production. 25 m long ropes were seeded maintaining same seeding gaps of 20 cm and average weight of each seed was 2 gm. Seeded ropes were transplanted in open sea with the help of bamboo poles maintaining a distance of 40 cm. Five floats were used in each rope to keep it floating during high tide. Seaweeds in the ropes were cultured for 30 days and then harvested. Fresh product of each rope was weighed separately. Dry yield was obtained by drying the fresh product whole day in open sun. Digital balance was used to measure the weight.

#### Experiment 3: effect of rope type

Two types of rope (where T_1_ denotes semi-floating single line method and T_2_ denotes semi-floating double line method) were followed to evaluate the effect of this method on the yield performance of *Gracilaria tenuistipitata.* “One-step” seeds were produced by cutting branched filamentous seaweeds into 5–10 cm long pieces. The cut pieces were attached to 25 m long ropes making holes by untwining at a gap of 20 cm. Seeded rope were then transplanted in open sea with the help of bamboo poles piled in the sand-flat which protected the ropes from being washed away by wave current. Ropes were placed at a distance of 40 cm. Plastic floats were used to keep the ropes hanging during high tide. Five floats were attached to each rope at a gap of 5 m. For semi-floating double line method, two adjacent single ropes were attached with bamboo sticks which gave the shape of a ladder. As average weight of each seed (2 gm.), seeding gap and length of ropes were same, therefore, initial inoculum of each rope was similar. Ropes were cultured for 30 days and then harvested. Fresh product of each rope was harvested separately, weighed with the help of digital balance and dried under open sun a whole day to obtain dry weight.

#### Experiment 4: effect of seeding gap

This experiment was carried out for determining the effect of seeding gap where T_1_,T_2_,T_3_ and T_4_ indicates 10 cm, 20 cm, 30 cm and 40 cm seeding gap, respectively on the yield performance of *Gracilaria tenuistipitata.* “One-step” seeds were produced by cutting branched filamentous seaweeds into 5–10 cm long pieces. Seeds were attached to ropes by making holes by untwining. Seeding gaps of ropes were different, i.e. 10 cm, 20 cm, 30 cm and 40 cm. Ropes were placed 40 cm apart from one another. Initial weight of each seed was 2 gm. Ropes were harvested 30 days after transplanting. Fresh product of each rope was harvested separately, weighed with the help of digital balance and dried under open sun a whole day to obtain dry weight.

### Statistical analysis

Data were statistically analyzed by statistical package SPSS version 16.0 (SPSS Inc., Chicago, IL, USA). Before all analysis data were analyzed for normality by probability plots and for homogeneity of variances by Levene's test. One way ANOVA was used to determine the significance of each parameter among different treatments. If a main effect was significant, the ANOVA was followed by Tukey's test. Level of significance was made at 95% probability level.
