# Author Correction: Yield improvement of *Gracilaria tenuistipitata* by optimizing different aspects in coast of cox’s bazar, Bangladesh

**DOI:** 10.1038/s41598-022-11718-3

**Published:** 2022-05-02

**Authors:** S. M. Bokhtiar, M. A. Ali, M. A. Z. Chowdhury, K. U. Ahmed, M. K. Hassan, M. Ahmed, M. S. Bhuiyan, O. F. Mashuk, M. M. Rahman, M. A. Salam, S. M. Rafiquzzaman, Md Faruque Hossain

**Affiliations:** 1 Bangladesh Agricultural Research Council, Dhaka, Bangladesh; 2 Bangladesh Agricultural Research Institute, Gazipur, Bangladesh; 3 Bangabandhu Sheikh Mujibur Rahman Agricultural University, Gazipur, Bangladesh

Correction to: *Scientific Reports* 10.1038/s41598-022-08040-3, published online 09 March 2022

Md Faruque Hossain was omitted from the author list in the original version of this Article.

The Author contributions section now reads:

“S.M.B., M.A.A., M.A.Z.C., K.U.A. designed and executed this research. M.K.H. and M.A. reviewed this manuscript. M.A.S designed and reviewed this manuscript. M.S.B., O.F.M., and M.M.R. performed research design, data compilation, manuscript preparation and draft manuscript preparation. S.M.R. supervised research activities. M.F.H. was actively engaged as co-principle investigator in execution of this research. All authors reviewed the manuscript.”

The original Article has been corrected.

